# In situ glacial survival maintains high genetic diversity of *Mussaenda kwangtungensis* on continental islands in subtropical China

**DOI:** 10.1002/ece3.6768

**Published:** 2020-09-17

**Authors:** Miaomiao Shi, Yuyuan Wang, Tingting Duan, Xin Qian, Tong Zeng, Dianxiang Zhang

**Affiliations:** ^1^ Key Laboratory of Plant Resources Conservation and Sustainable Utilization South China Botanical Garden Chinese Academy of Sciences Guangzhou China; ^2^ Center of Conservation Biology Core Botanical Gardens Chinese Academy of Sciences Guangzhou China; ^3^ University of Chinese Academy of Sciences Beijing China; ^4^ Guangdong Ocean University Zhanjiang China; ^5^ College of Life Sciences Fujian Agriculture and Forestry University Fuzhou China

**Keywords:** continental islands, genetic diversity, in situ glacial survival, isolation, subtropical China

## Abstract

Generally, island populations are predicted to have less genetic variation than their mainland relatives. However, a growing number of studies have nevertheless reported exceptions, indicating that the relationships were impacted by several factors, for example, historical processes. In the present study, we chose a group of subtropical islands located in South China as the study system, which are quite younger and much closer to the mainland than most of the previous studied island systems, to test the hypothesis that in situ glacial survival contributes to high levels of genetic diversity in island populations. We conducted a comparison of genetic variation between 12 island and 11 nearby mainland populations of *Mussaenda kwangtungensis* using eleven nuclear microsatellite and three chloroplast markers, evaluated effects of the island area and distance to mainland on genetic diversity of island populations, and simulated the potential distribution over the past by ecological niche modeling, together with the genetic data to detect the role of islands during the glacial periods. The island populations displayed comparable levels of genetic diversity and differentiation with mainland populations, overall high levels of unique polymorphisms, and the greatest values of specific within‐population genetic diversity. No significant correlation was detected between genetic diversity of island populations and distance to mainland, as well as area of islands, except that allelic richness was significantly positively correlated with the area of islands. Nuclear microsatellites revealed two main clusters, largely corresponding to islands and inland populations, which divergence dated to a time of island formation by ABC analysis. Ecological niche modeling predicted a highly climatic suitability on islands during the last glacial maximum (LGM). Our results suggest that the islands have acted as refugia during the LGM and highlight the role of in situ glacial survival in maintaining high levels of genetic diversity of *M. kwangtungensis* in continental islands of subtropical China.

## Graphical abstract

We conducted a comparison of genetic variation between island and mainland populations of *Mussaenda kwangtungensis* in a young and near mainland–island system. Our results suggest that the islands have acted as refugia during the LGM and highlight the role of in situ glacial survival in maintaining high levels of genetic diversity of *M. kwangtungensis* in continental islands of South China.

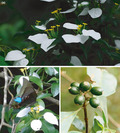

## 1 INTRODUCTION

Islands are often regarded as natural laboratories and play key roles in the study of ecology and evolution (Adsersen, 1995; Crawford & Stuessy, 1997; MacArthur & Wilson, 1967; Warren et al., 2015). Indeed, since Darwin's time, island ecosystems have received a great deal of attention from ecologists and evolutionary biologists. Islands possess a series of unique characteristics; their small size, distinct boundaries, and simplified biotas mean that it is easier to observe and interpret patterns of evolution and population genetic structure in these settings (Losos & Richlefs, 2009). The natural geographical barriers of oceans also restrict gene flow between islands and the mainland or among islands; this provides opportunities to infer the genetic consequences of fragmentation or isolation as well as population differentiation (Liu, Zhang, Chen, Compton, & Chen, 2013; Wang et al., 2014).

We know that isolated populations of plant species are susceptible to environmental and demographic stochasticity (MacArthur & Wilson, 1967). As a result of this lack of connectivity, isolated island populations suffer from a multitude of demographic and genetic consequences, including a loss of genetic diversity (Frankham, 1997), increased inbreeding and likelihood of extinction (Lande, 1994). The loss of genetic diversity on islands can be primarily attributed to a small number of founders for population establishment at small sizes (Maki, 2001). Thus, assuming that species colonize to islands from the mainland under a stepping‐stone model, less isolated, large, nearshore islands may serve as intermediate stepping stones for the colonization of more distant islands. Intense isolation effects can also be expected on distant islands; those that are situated close to a mainland source therefore have a greater potential for repeated colonization (Wallace, Wheeler, McGlaughlin, Bresowar, & Helenurm, 2017). This means that these areas have a chance of greater diversity (Kimura & Weiss, 1964). In general, genetic variation decreases negatively with effective population size (Wright, 1978); genetic diversity can therefore be predicted to fall much more rapidly on smaller islands because of their vulnerability to demographic stochasticity and drift (Wright, 1978). Limited gene flow impeded by geographical isolation in many cases can also lead to lower levels of island genetic variability (Frankham, 1997). Island populations also generally contain less genetic variation than their continental relatives. Although this hypothesis has been confirmed in several island systems (García‐Verdugo et al., 2009; Matsumura, Yokoyama, Fukuda, & Maki, 2009), a growing number of studies have nevertheless reported exceptions. In one example, Chen, Shi, Ai, Gu, and Chen (2008) demonstrated similar genetic variation in island and mainland populations of *Ficus pumila* due to high gene flow. A similar pattern was also revealed in *Cryptomeria japonica*, attributed to islands acting as refugia for this species during the last glacial maximum (LGM) (Tsumura & Ohba, 1993). Additionally, Fernández‐Mazuecos and Vargas (2011) detected more diversity in islands than in mainland populations of *Cistus monspeliensis*, something which can be explained by genetic bottlenecks on the continent during glaciations in the Quaternary. These case‐specific studies indicate that a lower level of genetic variation on islands is not an absolute rule and that life‐history traits, island age, isolation, and historical demography may also be significant predictors of this variable.

Historical processes, especially drastic Quaternary climatic fluctuations, have resulted in multiple contraction–expansion processes. These have long been considered to have exerted profound effects on the geographical distribution and current genetic structure of extant species (Hewitt, 2004; Hickerson et al., 2010; Qiu, Fu, & Comes, 2011; Shi, Michalski, Welk, Chen, & Durka, 2014). Islands are often characterized by topographical and environmental heterogeneities and tend to have more stable climates relative to mainland areas (Weigelt, Jetz, & Kreft, 2013). This means that these regions have great potential to serve as efficient refugia for plant species during glacial periods. Genetic information also provides novel insights on past vegetation history and gives us the chance to address important paleoecological questions (Petit et al., 2003). Accumulating genetic evidence has implied a role for refugia during glacial periods in different island systems, including the Canary Islands (Condamine, Leslie, & Antonelli, 2017; Fernández‐Mazuecos & Vargas, 2011; García‐Verdugo et al., 2013; Hutsemékers et al., 2011), Ryukyu Archipelago (Nakamura et al., 2014), and on Australian islands (Gibson et al., 2017). Long‐term studies based on these oceanic islands have greatly advanced our understanding of complex biogeography. Understanding, however, remains limited with regard to younger, continental shelf islands as these have remained isolated since the submergence of Pleistocene land bridges and have therefore long been thought to possess relic biotas of limited evolutionary importance. Phylogeographic studies on continental islands seek explanations regarding the demographic history of populations (Nakamura et al., 2010) and therefore provide powerful tools to interpret current genetic structures versus the mainland.

China has more than 10,000 islands, including mostly continental examples (>90%) with climates ranging from tropical to cold temperate (Committee, 2013; Wang et al., 2014). Subtropical China (ca. 34°N to 22°N), characterized by evergreen broad‐leaved forests (Wu, 1980), has never been covered by large ice sheets during the LGM. Climate here was cooler by 4–6°C at this time and markedly drier by 400–600 mm/year (Qiu et al., 2011). Sea level fluctuations in the Pleistocene resulted in recurring land bridges connecting the southern China mainland and continental islands, which may have provided suitable habitats for species to migrate and survive (Jiang, Gardner, Meng, Deng, & Xu, 2019). Simulated vegetation maps have indicated that land bridges in South China were occupied by tropical forests and nonforest when climate cooled during the LGM (Harrison, Yu, Takahara, & Prentice, 2001). To date, however, limited phylogeographic evidence reveals the evolution of species with disjunct distributions on islands and the mainland because of less emphasis and inadequate sampling, even though such studies in subtropical China have increased rapidly in recent years (Chen et al., 2020; Gong et al., 2016; Qiu et al., 2011; Shi et al., 2014).

Guangdong Province is located on the southernmost of Mainland China and boasts a 2,414 km coastline that includes the transition from the southern subtropical to tropical zone. This area encompasses *ca*. 750 continental islands (>500 m^2^) with an area of about 1,592 km^2^ (Li & Zhou, 2010). The bulk of these islands are close to the mainland (<30 km), especially those located off the Pearl River Mouth. The depth of the sea among islands ranges between 20 m and 30 m (Chen, Tong, & Mao, 1993). Indeed, during the LGM (13,700 years ago ± 600 years), sea level dropped to its lowest position, 131 m lower than current, forming a large area of land bridges. Sea level then rose rapidly in the Holocene, from −131 m to −40 m, reaching a postglacial maximum of between 3 m and 5 m higher than today around 6,000–7,000 years ago and fell to its present position until 5,000 years ago (Chen et al., 1993; Chen, Bao, Chen, & Zhao, 1990). Quaternary sporopollen assemblages show that this region was mainly covered by mixed northern subtropical evergreen and deciduous broad‐leaved forests (Chen et al., 1993). These indicate that islands off the Pearl River Mouth served as glacial refugia during the LGM and there are chances of in situ glacial survival within extant island populations.

The climbing shrub *Mussaenda kwangtungensis* H. L. Li (Rubiaceae) is endemic to Guangdong Province and has a very narrow distribution. This plant occurs only in the southern part of Guangdong Province, commonly on islands and the adjacent mainland. This species is placed within a very young genus which derived in the Miocene (7.82–14.44 Ma) (Duan et al., 2018) but that which then underwent rapid diversification from 4 Ma onwards. The clade containing *M. kwangtungensis* also has a very short evolutionary history (<1 Ma). We report here a comparison of genetic diversity between 12 island populations and 11 adjacent mainland populations of *M. kwangtungensis* carried out using 11 nuclear microsatellite and three chloroplast markers. Data enabled us to detect the impacts of isolation and demographic history on the genetic structure of *M. kwangtungensis*. Thus, by combining approaches from molecular phylogeography and ecological niche modeling, we tested the hypothesis that in situ glacial survival contributes to high levels of genetic diversity in island populations. We addressed the following questions: (a) Do island populations have lower genetic diversity compared to mainland populations following general expectations? (b) Is genetic diversity significantly correlated with island characteristics such as area and distance to mainland? (c) Did island regions likely act as refugia for *M. kwangtungensis* during the LGM? (d) What is the demographic history of the existing *M. kwangtungensis* populations?

## 2 MATERIALS AND METHODS

### 2.1 Species and sampling locations

**Figure 1:**
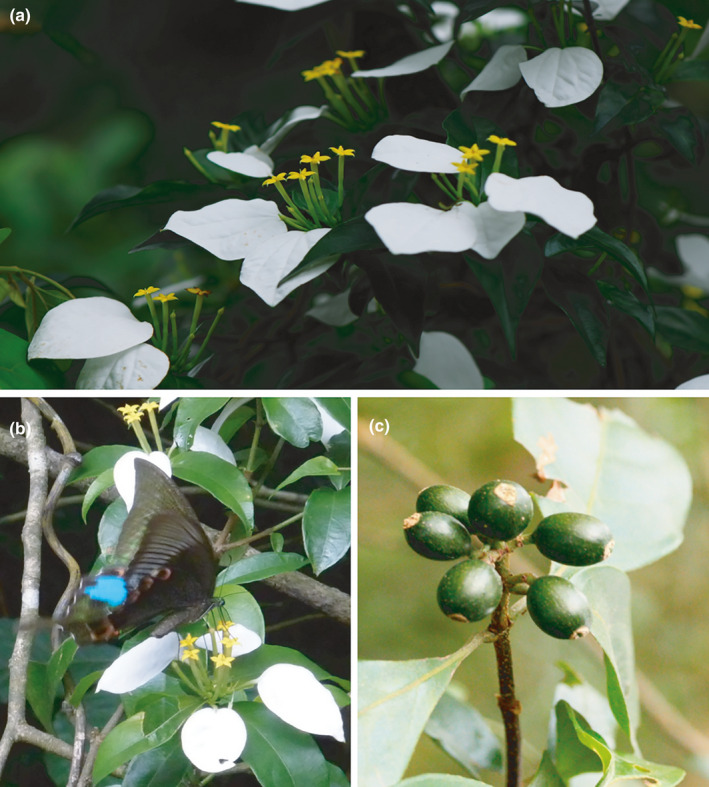
(a) *Mussaenda kwangtungensis*. (b) *Papilio paris* visiting the flowers of *M*. *kwangtungensis*. (c) Fruits of *M*. *kwangtungensis*

**Figure 2:**
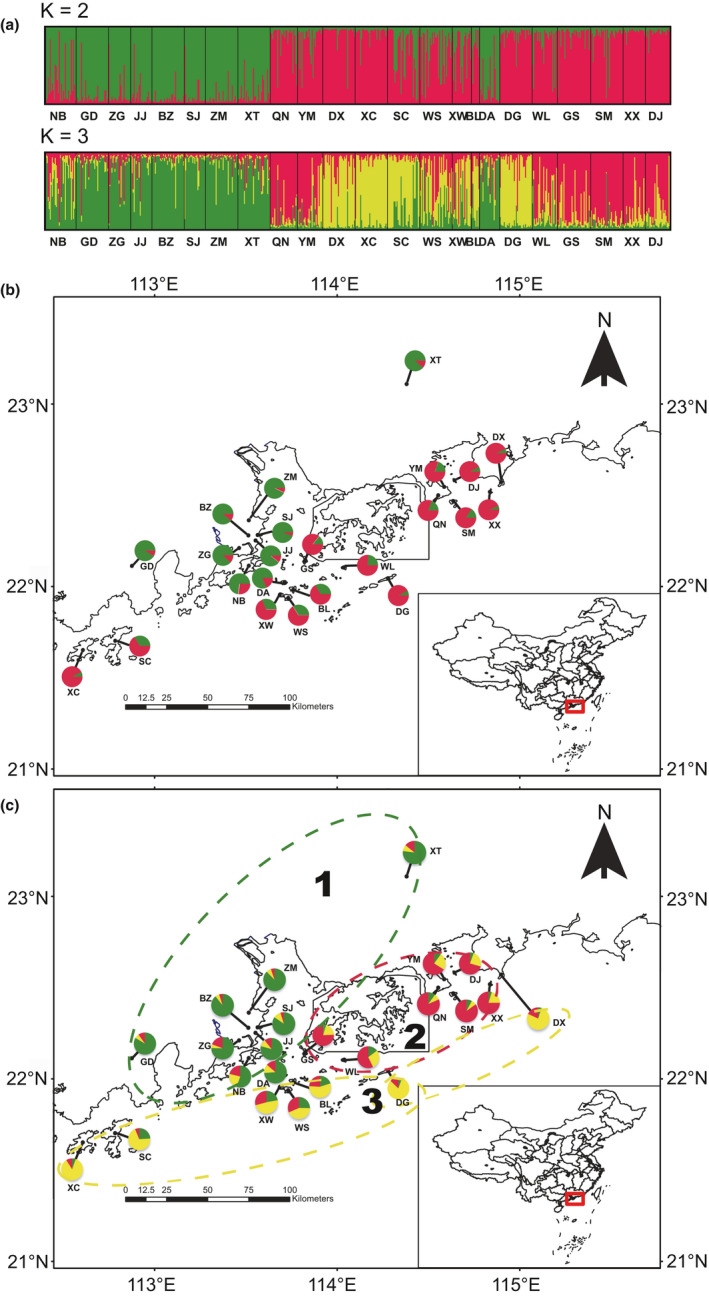
(a) Genetic clusters of 23 *Mussaenda kwangtungensis* populations assigned by STRUCTURE. Each individual is represented by a vertical bar, partitioned into *K* = 2 and *K* = 3 clusters. Map showing the distribution and genetic structure of populations for the most likely *K* = 2 (b) and *K* = 3 (c). Colors of dashed ellipses correspond to the three groups classified according to where populations are located and the STRUCTURE clustering results when *K* = 3. Number in ellipses marks the corresponding gene pool used for ABC analysis

*Mussaenda kwangtungensis* H. L. Li (Rubiaceae) is a perennial, climbing shrub, between 1 m and 2.5 m in height. It has papery leaves with 3–9 cm × 1–3 cm in size and lanceolate‐elliptic to elliptic‐oblong in shape. Its inflorescences are compact cymose‐to‐subcapitate, and each possesses white, blade oblong‐ovate, elliptic, or elliptic‐ovate petaloid calycophylls with 3.5–5 cm long and 1.5–2.5 cm wide (Figure 1). The tubular flowers are yellow and 25–35 mm in length. This species flowers between May and September, with a peak between June and July; berry fruits mature between May and November (Luo et al., 2015). *M. kwangtungensis* is a typical functional dioecious species (Duan et al., 2018; Li, Wu, Zhang, & Barrett, 2010) which is pollinated by butterflies, moths, and bees (Luo et al., 2015). This species is endemic to China; it has a very narrow distribution, only occurring in South Guangdong Province (Figure 2) in secondary, disturbed forests, in open habitats, and on sunny slopes.

The study region assessed here encompasses the whole distribution range of *M*. *kwangtungensis*, comprising offshore islands in Guangdong Province, South China, as well as adjacent mainland areas (Figure 2). We collected samples from 23 populations (Table 1), including 12 island populations from 12 islands, alongside eight inland and three peninsula populations. The area of selected islands varies from 1.27 to 157 km^2^ (Committee, 2013), and the nearest distance between an island and the mainland fell within a range of 3.8 to 77.7 km (Table S1 in Appendix S1). In each population, between eight and 33 individuals (mean = 27) were sampled, a total of 617 individuals. Each sample was separated from others by at least 30 m in order to reflect the real pattern of the population as much as possible. About five healthy leaves were collected from each individual and dried using silica gel. The voucher specimen for each population was deposited in South China Botanical Garden, Chinese Academy of Sciences (Table S2: Appendix S1).

**Table 1:** Population code, sample size, and genetic characteristics of *Mussaenda kwangtungensis*

Population code	SSR					cpDNA			
	*N*	*N*_A_	*A*_R_	*H*_O_	*H*_E_	*n*	Haplotypes	*h*	*π* × 10^–3^
**Mainland_inland**									
NB	30	9.3	5.82	0.503	0.704	6	H3 (1), H6 (4), H7 (1)	0.600	0.398
GD	32	10.1	6.03	0.640	0.743	8	H1 (4), H6 (2), H10 (2)	0.714	0.244
ZG	22	8.7	6.26	0.522	0.748	5	H1 (1), H7 (4)	0.400	0.114
JJ	21	8.4	5.92	0.547	0.748	7	H1 (5), H8 (1), H9 (1)	0.524	0.325
BZ	32	8.5	5.58	0.602	0.728	4	H1 (4)	0	0
SJ	21	7.5	5.58	0.608	0.727	6	H1 (4), H3 (1), H8 (1)	0.600	0.398
ZM	32	10.1	5.93	0.680	0.715	6	H1 (2), H3 (1), H4 (2), H5 (1)	0.867	0.569
XT	32	9.5	5.69	0.568	0.704	6	H2 (5), H3 (1)	0.333	0.190
**Mainland_peninsula**									
QN	27	9.4	5.89	0.634	0.711	5	H1 (3), H7 (2)	0.600	0.171
YM	25	10.1	6.39	0.618	0.744	4	H2 (1), H11 (1), H12 (2)	0.833	1.327
DX	32	9.2	5.38	0.494	0.636	3	H3 (1), H7 (2)	0.667	0.190
**Island**									
XC	32	9.3	5.91	0.619	0.725	2	H13 (1), H14 (1)	1.000	1.137
SC	32	10.7	6.62	0.591	0.745	3	H7 (3)	0	0
WS	32	8.6	5.53	0.593	0.693	4	H1 (1), H3 (1), H8 (1), H16 (1)	1.000	0.948
XW	19	7.1	5.30	0.599	0.682	8	H3 (2), H8 (1), H16 (1), H17 (2), H18 (2)	0.893	0.670
BL	8	5.7	5.73	0.602	0.757	3	H7 (3)	0	0
DA	20	7.8	5.67	0.600	0.711	7	H1 (1), H16 (5), H21 (1)	0.524	0.731
DG	32	8.6	5.32	0.459	0.602	4	H2 (4)	0	0
WL	25	7.5	5.01	0.457	0.622	4	H1 (4)	0	0
GS	33	8.0	5.30	0.496	0.678	7	H1 (2), H7 (5)	0.476	0.135
SM	32	9.5	5.98	0.552	0.712	5	H1 (5)	0	0
XX	22	8.1	5.25	0.437	0.657	3	H15 (3)	0	0
DJ	24	8.8	5.73	0.576	0.696	5	H12 (2), H19 (2), H20 (1)	0.800	0.967
Mean	26.8	8.7	5.73	0.565	0.704	5.0		0.471	0.370
Overall	617	21.6	6.88	0.566	0.705	115		0.855	0.619

Note

*N* and *n*, sample size used in microsatellite and cpDNA analysis, respectively; *N*_A_, number of alleles per locus; *A*_R_, allelic richness based on 8 samples; *H*_O_, observed heterozygosity; *H*_E_, expected heterozygosity; *h*, haplotype diversity; and *π*, nucleotide diversity. Values in parentheses indicate the frequency of each haplotype.

### 2.2 DNA extraction, microsatellite genotyping, and chloroplast sequencing

Total genomic DNA of *M. kwangtungensis* was extracted from ca. 50 mg samples of silica gel‐dried leaves using the modified cetyltrimethylammonium bromide (CTAB) method (Doyle, 1991). Thus, 11 nuclear microsatellite loci (i.e., CT99, CT142, CT48, CT59, CAA92, CT135, CT17, AC30, CT113, CT12, and CT60) were amplified for all 617 individuals using fluorescently labeled primers (Duan & Zhang, 2014; Appendix S1). Alleles were then scanned on ABI Prism 3100 Genetic Analyzer (Invitrogen, China) with internal size standard GeneScan™ 500 LIZ. and called by GeneMarker v. 2.4.0 (Holland & Parson, 2011). In order to minimize the genotyping error generated during different runs (Hoffman & Amos, 2005), the same laboratory methods, personnel, sequencing instrument, and allele bins in GeneMarker were used in all cases. Additionally, around 60 individuals were randomly selected as replicates, which were amplified and scanned at least twice in different runs.

For chloroplast sequencing, between two and eight individuals (mean = 5) in each population were successfully sequenced for three chloroplast intergenic spacer regions: ndhf‐rpl32, psbE‐petL, and rpl32‐trnL (Shaw, Lickey, Schilling, & Small, 2007). Polymerase chain reactions (PCRs) were conducted following the protocols outlined in Appendix S1. PCR products were then sequenced by an ABI 3,730 automated DNA analyzer (Applied Biosystems). DNA sequences were assembled using Sequencher 5.3 (Gene Codes Corporation 2015), aligned with BioEdit 7.1.11 (Hall, 1999) and deposited in GenBank, under accession numbers MT349425–MT349442 and MT361334–MT361343.

### 2.3 Nuclear data analysis

#### 2.3.1 Genetic diversity and differentiation

To address the effectiveness of the microsatellite loci, we performed three analyses. First, linkage disequilibrium among loci per population was tested using FSTAT 2.9.3 (Goudet, 1995). Second, population‐specific tests of deviation from Hardy–Weinberg equilibrium were conducted with GenAlEx 6.5 (Peakall & Smouse, 2006). All multiple tests were adjusted by sequential Bonferroni correction (Rice, 1989). Third, the presence of null alleles was checked using MICRO‐CHECKER (van Oosterhout, Hutchinson, Wills, & Shipley, 2004).

Genetic diversity at both species and population levels was characterized by calculating the number of alleles (*N*_A_), allelic richness (*A*_R_, correcting for sample size by rarefaction), and observed (*H*_O_) and unbiased expected heterozygosity (*H*_E_). Analyses were performed using FSTAT. Further, in order to investigate differences in genetic diversity between mainland (inland + peninsula) and island populations, we compared *A*_R_, *H*_O,_ and *H*_E_ between the two groups by permuting populations 1,000 times using FSTAT. In order to check the correlation of genetic diversity with island characteristics, we conducted linear regressions between genetic diversity of island populations and island characteristics (area and the nearest distance to mainland, both of which were transformed by natural logarithm to fit a normal distribution) using R 3.3.1 (R Development Core Team, 2012).

Overall and pairwise population differentiation was assessed by *F*_ST_ (θ) using FSTAT. Standard error was estimated by Jackknifing for overall *F*_ST_, and 1,000 bootstraps were used to estimate the 95% confidence interval. As *F*_ST_ is likely to underestimate genetic differentiation between populations for markers which show high levels of allelic variability, we also calculated *F'*_ST_, a standardized parameter of genetic differentiation as *F'*_ST_ = *F*_ST_/*F*_STmax_ (Hedrick, 2005). *F*_STmax_ was calculated after recoding the data using RECODEDATA (Meirmans, 2006). To test whether genetic differentiation was higher among island populations than that among mainland populations, differences among mean pairwise *F*_ST_ values were evaluated using a randomization procedure with 1,000 permutations in FSTAT. To determine the level of genetic differentiation between the mainland and island populations, an analysis of molecular variation (AMOVA) was carried out using ARLEQUIN 3.5.2.2 (Excoffier & Lischer, 2010). We also assessed patterns of isolation by distance using Mantel tests, based on the regression of pairwise estimates of genetic distances (*F'*_ST_/(1 − *F'*_ST_)) against corresponding logarithmic (log_10_) geographical distances for all populations and two groups. These analyses were performed with the R package “ecodist” (Goslee & Urban, 2007) with 10,000 permutations to determine statistical significance.

#### 2.3.2 Genetic clustering

Population clusters and ancestry were detected by Bayesian clustering using STRUCTURE 2.3.3 (Pritchard, Stephens, & Donnelly, 2000), which makes use of Markov chain Monte Carlo (MCMC) simulations to infer the proportion of ancestry of genotypes from *K* distinct clusters. An admixture model was run with correlated allele frequencies. Each run was pursued for 500,000 MCMC interactions, with an initial burn‐in of 100,000. To estimate the *K* number of ancestral genetic populations and the ancestry membership coefficients of each individual in these clusters, the algorithm was run 10 times for each user‐defined *K* value from 1 to 10. The log‐likelihood value Ln P(*K*) and delta *K* (Δ*K*) were calculated to determine the most likely number of clusters, as suggested by Evanno, Regnaut, and Goudet (2005), using a web‐based program STRUCTURE HARVESTER (Earl & vonHoldt, 2012). Replicates for the best number of clusters were compared to identify optimal clustering scenarios with CLUMPAK (Kopelman, Mayzel, Jakobsson, Rosenberg, & Mayrose, 2015). Finally, the corresponding Q matrices were visualized using DISTRUCT (Rosenberg, 2004).

#### 2.3.3 Demographic analysis

Demographic history of populations that had experienced a recent (past 2*N*_e_–4*N*_e_ generations) reduction in effective population size was detected using BOTTLENECK based on microsatellite data (Piry, Luikart, & Cornuet, 1999), which assumes that alleles are generally reduced faster than heterozygosity; thus, populations experiencing recent bottlenecks will display an excess of heterozygosity relative to that expected from the equilibrium between mutation and drift (Piry et al., 1999). Two models were selected, a stepwise mutation model (SMM) and a two‐phase model (TPM), believed to be appropriate for microsatellite evolution (Luikart & England, 1999). The latter, TPM, was set with 70% SMM characters, while significance was estimated by Wilcoxon sign‐rank test based on 1,000 replications. We also tested the presence of mode‐shifts from the normal L‐shaped distribution of allelic frequencies, typical of an equilibrium situation in stable populations (Luikart, Sherwin, Steele, & Allendorf, 1998).

Approximate Bayesian computations (ABC) are powerful tools for inference on population divergence and demographic history and thus have been frequently used in population genetics and phylogeographic studies (Aoki et al., 2019; Zeng, Wang, Liao, Wang, & Zhang, 2015). We used software DIYABC v.2.0.4 to test alternative hypotheses for the history of divergence of genetic clusters (Cornuet et al., 2014). Since STRUCTURE identified *K* = 2 as the most appropriate number of clusters, followed by a second peak at *K* = 3, we defined three gene pools on the basis of these results. Thus, each population, except for the DA population, was assigned to one of the three gene pools corresponding to its largest proportion of ancestry (Figure 1). The DA population was excluded from analysis because it was the only island population mainly constituted by mainland cluster. As a result, gene pool 1 consisted of 222 individuals from inland region; gene pool 2, of 188 individuals from peninsular and island regions; and gene pool 3, of 187 individuals also from peninsular and island regions. By comparing the posterior probabilities of seven scenarios of population divergence and admixture among these three gene pools (Table 2, Fig. S1 in Appendix S2), we tested the following alternative hypotheses for *M. kwangtungensis* glacial refugia: the presence of just one refugium, located either in inland (scenario 1) or in island (scenario 2); the presence of two refugia located in inland and island, respectively (scenario 3–6); and the presence of three refugia, one located in inland and two in islands (scenario 7). Prior values used for all parameters are listed in Table S3 (Appendix S2). Summary statistics were calculated for each scenario by selecting the scenario and estimating the posteriors of demographic parameters. Seven million simulations were run in total, of which the 1% closest to the observed data were used to estimate the relative posterior probabilities of each scenario with a logistic regression approach. The relative likelihoods of the seven scenarios were compared by logistic regression on 1% of the closest simulated data points.

**Table 2:** Description of the seven scenarios in ABC analysis for divergence and admixture history of *Mussaenda kwangtungensis*

Scenario	Description	Refugial location
One refugium		
Scenario 1	Ancestral effective population size varied at t2, and gene pools 2 and 3 arose *via* divergence from gene pool 1 at t1	Inland
Scenario 2	Ancestral effective population size varied at t2, and gene pools 1 and 3 arose *via* divergence from gene pool 2 at t1	Island
Two refugia		
Scenario 3	Ancestral effective population size varied at t3. Gene pools 1 and 2 diverged at t2, and then gene pool 3 arose *via* divergence from 2 at t1	One in inland and One in island.
Scenario 4	Ancestral effective population size varied at t3. Gene pools 1 and 3 diverged at t2, and then gene pool 2 arose *via* divergence from 3 at t1	One in inland and One in island.
Scenario 5	Ancestral effective population size varied at t3. Gene pools 1 and 2 diverged at t2, and then gene pool 3 arose *via* an admixture event of parental demes 1 and 2 at t1	One in inland and One in island.
Scenario 6	Ancestral effective population size varied at t3. Gene pools 1 and 3 diverged at t2, and then gene pool 2 arose *via* an admixture event of parental demes 1 and 3 at t1	One in inland and One in island.
Three refugia		
Scenario 7	Ancestral effective population size varied at t2, and gene pools 1, 2, and 3 diverged simultaneously at t1	One in inland, and two in islands

#### 2.3.4 Contemporary and historical gene flow

To evaluate the migration rates over contemporary and historical timescales, we used the programs BAYESASS (Wilson & Rannala, 2003) and MIGRATE (Beerli, 2008), respectively. Both analyses were conducted at regional scale based on the two clusters identified by STRUCTURE. In BAYESASS analysis, each run performed for 1 × 10^7^ iterations with the chain sampled every 2,000 iterations. A burn‐in of 10^6^ was used, and delta values were adjusted to ensure that 40%–60% of the total changes were accepted. As recommended in a recent evaluation of BAYESASS (Faubet, Waples, & Gaggiotti, 2007), 10 multiple runs were performed using different initial seeds. We presented the results for the run displaying the best model fit as indicated by the Bayesian deviance measure (Spiegelhalter, 2002).

In terms of assessing historical migration rates, MIGRATE was run with subsets of the data (consisting of equal sizes of ~50) using default settings (10 short chains of 10^3^ sampled, 500 recorded, and three long chains of 10^4^ sampled, 5,000 recorded). Four‐chain heating at temperatures of 1.0, 1.5, 3.0 and 100,000 was implemented to increase the efficiency of the MCMC. We repeated the runs three times until posterior probabilities stabilized using prior estimates of θ (the relative effective population size, 4*N*_e_ *μ*) and *M* as starting parameters. Maximum‐likelihood estimates and confidence intervals were reported from the final run. To compare historical and contemporary migration rates generated by BAYESASS and MIGRATE, we used the values of *m* directly generated by the former and converted *M* values generated by the latter to *m* based on the formula *M* = *m*/*μ*, where *μ* is the mean mutation rate of microsatellites. The *μ* was set to 10^–4^–10^–3^, the same as that used in ABC analysis.

### 2.4 CpDNA sequence analysis

#### 2.4.1 Diversity and differentiation

Haplotypes were identified using DNASP 5.10 (Librado & Rozas, 2009). The phylogenetic relationships among haplotypes were visualized as a statistical parsimony network computed with TCS (Clement, Posada, & Crandall, 2000). We calculated haplotype diversity (*h*) and nucleotide diversity (*π*) per population using ARLEQUIN. The significance of differences in *h* and *π* between mainland and island populations was investigated by the *t* test at the 5% level (R Development Core Team, 2012). Correlations between diversity indices (no. of haplotypes, *h* and *π* for island populations) and island characteristics (natural logarithm of island area and the nearest distance to mainland) were tested using linear regression analyses performed with R 3.3.1. To access the genetic differentiation, we computed *F*_ST_ values for all populations and both groups (mainland and island) identified by cpDNA sequencing using ARLEQUIN. An analysis of molecular variation (AMOVA) was also carried out to determine the level of genetic differentiation between mainland and island populations in ARLEQUIN. We also calculated pairwise *F*_ST_ values and tested for isolation by distance by evaluating the significance of the correlation between genetic differentiation and log geographical distances.

#### 2.4.2 Demographic history

We used values of Tajima's *D* and Fu's *F*s to infer potential population growth and expansion using ARLEQUIN. It is clear that significantly negative *D* and *F*s values often indicate recent population expansion following a sever reduction in population size (Fu, 1997). Mismatch distribution analysis was then also conducted under the model of demographic expansion. The goodness of fit was tested with the sum of squared deviation (*SDD*) between observed and expected mismatch distribution, and Harpending's raggedness index (*H*_Rag_) using 1,000 parametric bootstrap replicates. The expansion time (*T*) for expanding groups was calculated based on the formula *T* = τ/2*μkg* (Rogers & Harpending, 1992), where *μ* is the substitution rate in substitution/site/year (s/s/y), *k* is the average sequence length used for analysis (3,530 bp in this study), and *g* is the generation time in years. A value for *μ* was set as 1.52 × 10^–9^ s/s/y, as proposed by Yamane, Yano, and Kawahara (2006) for noncoding chloroplast regions, with a confidence interval of 1.0–3.0 × 10^–9^ s/s/y (Wolf, Li, & Sharp, 1987). The value of *g* was approximated as 10 years in shrubs. We also used the Bayesian skyline plots (BSPs) method as implemented in BEAST v.2.6.2 (Bouckaert et al., 2014) to estimate fluctuations in effective population size using the above substitution rate. MCMC chains were run for 2 × 10^7^ under the GTR model selected by ModelFinder (Kalyaanamoorthy, Minh, Wong, von Haeseler, & Jermiin, 2017). The coalescent Bayesian Skyline model was used under a relaxed clock model with an uncorrelated exponential distribution. We summarized the posterior parameter distributions and calculated the parameter‐trace ESS with Tracer v.1.7.1. A BSP was then reconstructed for population changes based on the tree file. All analyses were performed for all populations, as well as the two clusters identified by STRUCTURE.

### 2.5 Ecological niche modeling

To identify the potential species distribution during the LGM, we calibrated climatic envelope models using georeferenced native occurrence records for the species and the Maxent algorithm. Current presence records of *M. kwangtungensis* were collected from the herbarium records and our sampled populations (Table S2). Bioclimatic variables were downloaded from the WorldClim database (http://worldclim.org) for three different periods: the last interglacial (LIG, ~120–140 kya), the LGM (~21 kya), and current. Palaeoclimatic layers were drawn both from the Community Climate System Model (CCSM) (Collins et al., 2006) and the Model for Interdisciplinary Research on Climate (MIROC) 3.2 in simulations of the potential habitats during the LGM. A cross‐validated model was then projected onto scenarios of LIG and LGM using MAXENT v.3.3.3 (Phillips, Anderson, & Schapire, 2006). In running models, we used the default values of convergence threshold (10^–5^) and the maximum number of iterations (500), using repeatedly randomized samples of 25% of localities for cross‐validation model training and testing, respectively. The regularization multiplier was set to 15 to fit smoother curves as these are thought to perform better when projected to different time periods, and because they do not overfit to training data. Model performance was evaluated using two different indices, the area under the curve (AUC) of the receiver operating characteristic and the true skill statistic (TSS). The predicted distribution at different periods was visualized on the map by ArcGIS v.10.2. Suitable areas for simulated models from MAXENT during different periods were calculated using ArcGIS by eligible considering distribution threshold greater than 0.5.

## 3 RESULTS

### 3.1 Nuclear genetic diversity and differentiation

Comparing with an adjusted P value, no linkage disequilibrium was found in any population among loci (*p* > .05) indicating that all 11 loci are independently inherited in *M*. *kwangtungensis*. Tests of deviation from the HWE, apart from CT12, revealed significant departures in four to 15 populations of other loci. Similarly, except for locus CT12, the other ten showed signals of null alleles in two to 15 populations with an average frequency between 3.15% and 13.83%. Analysis of genetic variation of *M*. *kwangtungensis* was therefore based on these 11 loci.

**Figure 3:**
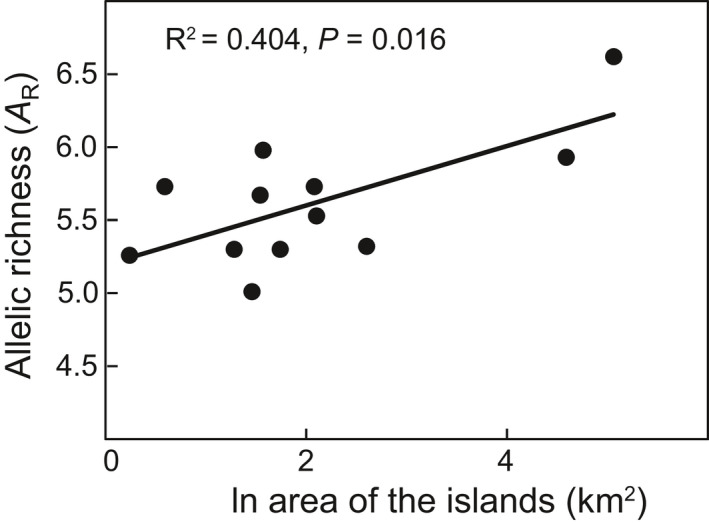
Relationship of allelic richness (*A*_R_) with area of the islands (km^2^) (transformation by natural logarithm)

At the species level, totally 239 alleles from 11 microsatellite loci were amplified with a mean of 21.6 per locus. Overall genetic diversity (*H*_E_) was 0.705. At the population level, the mean number of alleles (*N*_A_) across all 11 loci ranged from 5.7 (BL) to 10.7 (SC). When correcting for the sample size, allelic richness (*A*_R_) varied from 5.01 (WL) to 6.62 (SC). Observed heterozygosity (*H*_O_) ranged from 0.494 (DX) to 0.680 (ZM) and expected heterozygosity (*H*_E_) from 0.602 (DG) to 0.757 (BL) (Table 1). Of *A*_R_, *H*_O,_ and *H*_E_, none was significantly higher on the mainland than on islands (Table 3), indicating that genetic diversity was not significantly different between mainland and island populations. Correlating genetic diversity of island populations with the nearest distance to mainland showed no significant relationship (*p* > .08), while correlating with the area of islands, only allelic richness (*A*_R_) was significantly positively correlated (*p* = .016) (Figure 3).

**Table 3:** Comparison of microsatellite genetic diversity and differentiation within and among populations of *Mussaenda kwangtungensis* in mainland and islands

Parameters	Mainland	Island	*p*
*A*_R_	5.86	5.61	.141
*H*_O_	0.583	0.548	.360
*H*_E_	0.719	0.690	.160
*F*_ST_	0.073	0.067	.698

Note

*A*_R_, allelic richness based on 19 samples; *H*_O_, observed heterozygosity; *H*_E_, expected heterozygosity; *F*_ST_, Weir & Cockerham's genetic differentiation; *p* value indicates the significance between the mainland and island populations.

**Figure 4:**
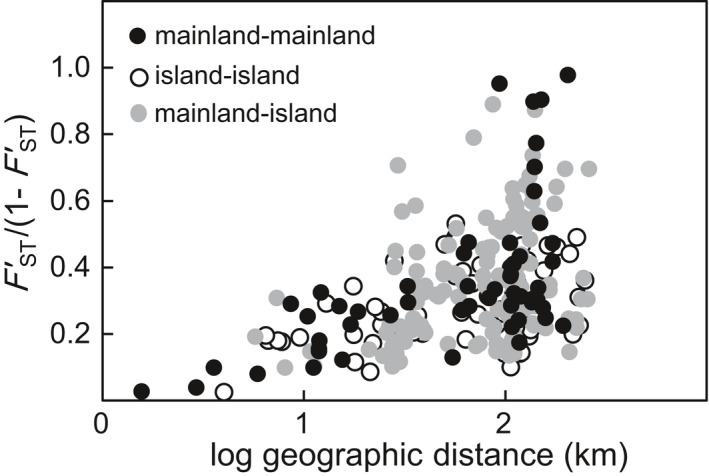
Patterns of isolation by distance in *Mussaenda kwangtungensis*. Pairwise genetic differentiation as a function of geographical distance based on microsatellites. Closed black dots: comparison between two populations from mainland; open dots: comparison between two populations from islands; closed gray dots: comparison between a population from mainland and an island population. All populations: *r* = 0.439, mantel *p* = .0001; mainland: *r* = 0.642, mantel *p* = .0011; islands: *r *= 0.323, mantel *p* = .0038

Overall *F*_ST_ across the 11 loci was 0.078 (*p* < .01), while standardized *F'*_ST_ was 0.247, indicating moderate differentiation. Genetic differentiation of mainland populations (*F*_ST_ = 0.073) was not significantly different compared to island populations (*F*_ST_ = 0.067) (*p* = .698) (Table 3). Our AMOVA revealed that only 0.90% of genetic variation resided among mainland and island populations (Table S4, Appendix S2). There was significant isolation by distance, as shown by the relationship of *F'*_ST_/ (1 − *F'*_ST_) with geographical distance (Mantel test) among all populations, as well as in both mainland and island populations (*p* < .05, Figure 4).

### 3.2 Nuclear genetic structure

Population structure and ancestry were inferred in STRUCTURE. The estimated logarithm of probability of the data, Ln P(*K*), increased slightly from *K* = 2, and the Δ*K* statistic was greatest for *K* = 2, with a second peak at *K* = 3 (Figure S2). At *K* = 2, all populations were divided into two clusters, geographically corresponding to inland and island plus peninsular regions, respectively. In terms of *K* = 3, inland cluster remained consistent, while DX, DG, XC, and SC were further clearly separated from island cluster, and WS, XW, and BL showed a large amount of mixture among gene pools (Figure 2).

### 3.3 Bottleneck and ABC analysis

Bottleneck analysis did not indicate excess of heterozygosity associated with recent bottlenecks in any mainland and island population, either under the SMM or TPM models (Table S5: Appendix S2). However, a shift from the normal L‐shaped distribution of allelic frequencies was found in the BL case.

A comparison of posterior probabilities of the seven scenarios using a logistic approach on 70,000 data points indicated that scenario 2 had the highest support (Figure S3), with a probability of 0.457 (95% CI: 0.365–0.549), associated with the hypothesis that there was one refugium in island and that mainland populations were derived from this area. Scenario 1 had the second highest support with a probability of 0.337 (95% CI: 0.229–0.447). Posterior probabilities for scenarios 3, 4, 5, 6, and 7 were 0.060, 0.053 0.023, 0.061, and 0.007, respectively. Posterior distribution of parameters for scenario 2 is shown in Table 4. Thus, if we consider a mean generation time of 10 years for *M*. *kwangtungensis*, the divergence of inland cluster (gene pool 1) from island cluster (gene pool 2), designated by t1, appears to have started 3,930 BP (95% CI: 1,020–7,140) compatible with the time of island formation during the Holocene. The median value of t2 (the time when ancestral effective population size varied) was 61,200 BP (95% CI: 8,120–96,700), indicating an increase of effective population size during the LGM (Table 4).

**Table 4:** Demographic approximate Bayesian computations for *Mussaenda kwangtungensis*, as obtained in the most likely scenario (scenario 2)

Parameter	Explanation	Median	q050	Q950
N1	Ne of gene pool 1	7,180	3,330	9,520
N2	Ne of gene pool 2	6,330	2,900	9,310
N3	Ne of gene pool 3	9,210	6,830	9,910
Na	Ne of ancestral gene pool 2	1,400	9.75	9,090
t1	Time to the divergence of 1 and 3 from 2	393	102	714
t2	Time of variation of ancestral Ne	6,120	812	9,670
μ	Mean mutation rate	7.64 × 10^–4^	4.03 × 10^–4^	9.78 × 10^–4^
p	Proportion of multiple step mutation in the generalized stepwise model	0.29	0.16	0.30
μ_SNI_	Mean single nucleotide insertion/ deletion rate	5.32 × 10^–6^	7.41 × 10^–8^	1.00 × 10^–6^

Note

*N*_e_ is the effective population size. q050‐q950 indicate the 95% credibility values.

### 3.4 Contemporary and historical gene flow

Based on BAYESASS analysis, the contemporary migration rate (*m*) from the mainland to islands was 0.005 (−0.002 to 0.013) while that from islands to the mainland was 0.016 (0.004–0.028). The overlapping 95% confidence intervals indicated no asymmetrical gene flow between island and mainland clusters. Similarly, MIGRATE did not reveal a pattern of asymmetrical gene flow between island and mainland. The relative migration rates (*M*) computed by MIGRATE were 4.450 (3.767–5.067) from the mainland to islands and 3.317 (2.533–4.033) in the other direction. When converting *M* to *m* for comparison, the median value of historical migration rate from the mainland to islands was 0.000445–0.00445 with *μ* ranging from 10^–4^ to 10^–3^, nonsignificantly different from contemporary migration rare, and vice versa.

### 3.5 CpDNA structure

**Figure 5:**
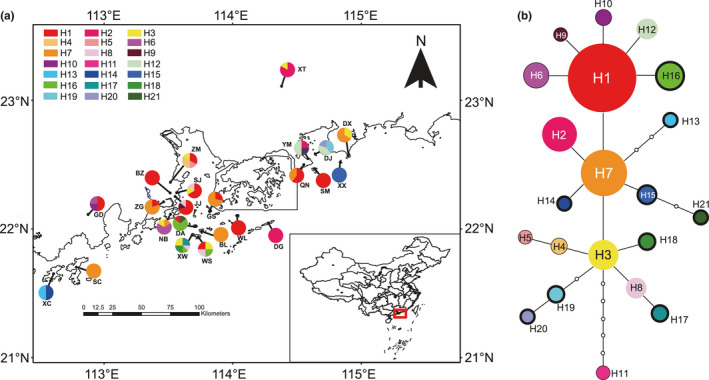
(a) The geographical distribution and respective frequency of 21 haplotypes from cpDNA sequencing in each population. Abbreviations of populations are given in Table 1. (b) The statistical parsimony network of cpDNA haplotypes. Size of circles indicates haplotype frequency. Open circles represent missing haplotypes. Thin and bold outlines indicate private haplotypes in mainland and islands, respectively, while circles without outlines are shared haplotypes

By aligning, *M*. *kwangtungensis* sequences yielded lengths of 920 bp, 1,224 bp, and 1,386 bp for rpl32‐trnL, psbE‐petL, and ndhf‐rpl32, respectively. A total of 28 variable sites, comprising 26 point mutations and two indels, were detected, defining 21 haplotypes (Table S6: Appendix S2). The geographical distribution of haplotypes is shown in Figure 5. The most widely distributed haplotype (H1) was found in 12 populations (52.2%) and 36 individuals, followed by H7 occurring in seven populations and 20 individuals. Six haplotypes (H1–3, H7, H8, and H12; 28.6%) were shared among mainland and island populations, while six (H4–6 and H9–11, 28.6%) and nine (H13–21, 42.9%) were privately restricted to mainland and islands, respectively. The parsimony network revealed closely related haplotypes (Figure 5b). The shared ones (H1, H7, and H3) were the most interior nodes and could be considered as ancestral. All the unique haplotypes in mainland and islands differed from them by one to three mutation steps, except for H11 by six steps from H3.

For *M*. *kwangtungensis*, within‐population haplotype diversity (*h*) was 0.471, and nucleotide diversity (*π*) was 0.370 × 10^–3^. On the mainland, each population had 1 (BZ) to 4 (ZM) haplotypes with a mean = 2.5. The value of *h* varied from 0 to 0.867 with a mean of 0.558, while *π* ranged from 0 to 1.327 × 10^–3^ (YM) (Table 1). On islands, the number of haplotypes per population varied from 1 (SC, BL, DG, WL, SM and XX) to 5 (XW), while values for *h* ranged from 0 to 1.000 (XC and WS) with a mean of 0.391 and *π* ranged from 0 to 1.137 × 10^–3^ (XC). None of diversity parameters (*h* and *π*) showed significant difference between mainland and island populations (*p* > .269). Correlation analysis between diversity at cpDNA (*h* and *π*) and island characteristics (area and distance to mainland) revealed no significant relationship (*p* > .160).

Population differentiation in cpDNA was revealed by *F*_ST_ = 0.402. Island populations showed a slightly stronger genetic differentiation (*F*_ST_ = 0.459) than their mainland counterparts (*F*_ST_ = 0.315). The results of AMOVA showed that just 0.34% of genetic variation existed among mainland and island groups (Table S4). No pattern of isolation by distance was revealed by Mantel tests in either all populations or within each group (*p* > .201).

### 3.6 Demographic history

At the species level, the neutrality test showed that both Tajima's *D* (−1.696, *p* = .012) and Fu's *F*s (9.885, *p* = .003) were negative. The mismatch distribution for haplotypes was unimodal with nonsignificant *SSD* and *H*_Rag_ (*p* > .05, Table 5), which was statistically consistent with the expansion model. Based on the corresponding τ value, the demographic expansion event was estimated to have occurred at 21,200 (10,700–32,200) years BP. Additionally, the BSPs analysis detected a slightly increasing population size until 20,000 years BP, then following a recent decrease (Fig. S4). When analyzed separately, island cluster showed the same pattern: negative *D* (−1.420, *p* = .052 and *F*s (−5.133, *p* = .022), unimodality of mismatch distribution (Table 5), and a slightly ascending curve followed by a fall for BSP (Fig. S4). The expansion event was dated at 17,000 (8,640 – 25,900) years BP. No signature of demographic expansion was detected in the case of the inland cluster. Both *D* (1.032, *p* = .810) and *F*s (1.723, *p* = .837) were positive. The mismatch distribution was bimodal with significant *SSD* (Table 5). BSP showed a very slightly ascending trend (Figure S4).

**Table 5:** Mismatch distribution analysis of cpDNA sequence data for *Mussaenda kwangtungensis*

Regions	τ	*T* (years)	Observed modality	*SSD*	*p*	*H*_Rag_	*p*
All	2.271	21,200 (10,700–32,200)	Unimodal	0.001	.655	0.027	.621
Inland	2.590	NC	Bimodal	0.204	.026	0.787	.270
Island	1.829	17,000 (8,640–25,900)	Unimodal	0.004	.501	0.026	.597

Note

Goodness of fit of observed to theoretical mismatch distributions under a demographic expansion model is tested with the sum of squared deviations (*SSD*) and raggedness index (*H*_Rag_). Confidence interval of expansion time *T* (in years BP) is shown in parentheses. NC, not calculated.

### 3.7 Ecological niche modeling

**Figure 6:**
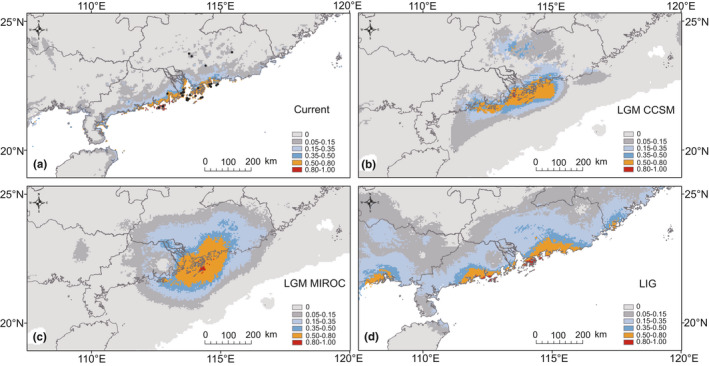
Modeled climatically suitable areas for *Mussaenda kwangtungensis* at different times. (a) Current; (b) the last glacial maximum (LGM, ~21 kya) under the CCSM (b) and MIROC (c) models; and (d) last interglacial (LIG, ~120–140 kya). The logistic value of habitat suitability is shown according to the color‐scale bars

The prediction of the bioclimatically suitable areas for *M*. *kwangtungensis* during the LIG, the LGM, and current is shown in Figure 6. The models in each period were indicated to have high predictive abilities with AUC (>0.99) and TSS (0.71–0.85). It is clear that *M*. *kwangtungensis* has been narrowly distributed at all stages. In general, the predicted distribution under current conditions was similar to the actual distributions. CCSM model suggested the habitats available during the LGM were relatively limited compared with the MIROC model. However, both of them showed the same trend: *M*. *kwangtungensis* experienced a recently spatial expansion when temperature decreased during the LGM period. In particular, the area with climatically suitability >0.5 enlarged from the present distribution to the southern ancient coast. Thus, by calculating the area with climatic suitability >0.5, larger region was highly suitable for *M*. *kwangtungensis* during the LGM based on CCSM (17,183 km^2^) and MIROC (29,560 km^2^) models, compared with that at present (10,118 km^2^). The area with climatic suitability >0.5 from the LIG (18,433 km^2^) was a little larger than that during the LGM based on the CCSM model, but far smaller than that from LGM based on MIROC model. Data show that when climatic suitability was greater than 0.8, CCSM model revealed greater area in inland than on islands, while in MIROC model, more suitable habitats were located on islands.

## 4 DISCUSSION

### 4.1 Similar levels of genetic diversity between mainland and island populations

Plastid and nuclear markers reveal high levels of genetic diversity in this species. Indeed, haplotype diversity based on cpDNA (*h*_T_ = 0.855) was higher than the mean value of angiosperms (*h*_T_ = 0.68) (Petit et al., 2005), while the expected heterozygosity computed on microsatellites (*H*_E_ = 0.705) was higher than values reported for narrow‐distributed/endemic species (*H*_E_ = 0.56/0.42) (Nybom, 2004). Life‐history traits, especially mating systems, may determine genetic structure among populations (Duminil et al., 2007). Species with mixed or outcrossing mating systems was demonstrated to have significantly higher genetic diversity than selfing species (Nybom, 2004). It is clear that *M. kwangtungensis* is a typical functional dioecious species with an obligate outcrossing mating system (Li et al., 2010), thus considered capable of harboring high genetic diversity (Hamrick & Godt, 1996). This has been evidenced by many previous studies (Shi & Chen, 2012; Shi et al., 2014).

Island populations show comparable genetic variation with mainland populations (Table 3), inconsistent with the general expectation that these tend to have lower genetic variation due to founder effects and geographical isolation (Frankham, 1997). We discuss possible explanations for this as similar, or higher, levels of genetic variation on islands have been variably ascribed to a number of variables including high gene flow. In this first case, we know that *M. kwangtungensis* is pollinated predominantly by butterflies (Papilionidae: *Papilio paris*, Figure 1) (Luo et al., 2015), in which long‐distance dispersals have been frequently observed (up to 13 km) (Baguette, 2003; Li, Zhang, Settele, Franzén, & Schweiger, 2013). Individuals of *P. paris* have been estimated to move up to 6 km (personal communication with Dr. X. Li), a distance that ensures pollen can be transferred between most islands and adjacent populations (Table S1). In addition, the color of berry fruits turns to purple black when they mature and attracts foraging birds (personal observation), which may provide opportunities for the long‐distance dispersal of seeds. At the same time, however, the high levels of private haplotypes on islands (Figure 5) also seem to indicate a lower level of occurrence while similarity in contemporary and historical gene flow indicate that isolation does not exert an influence on genetic structure (Chiucchi & Gibbs, 2010). Considering a relatively continuous distribution on the mainland, sufficient dispersal across the ocean barrier could be expected. Secondly, genetic bottlenecks on the mainland might provide another explanation as these have occurred in the recent past due to human disturbance. The construction of buildings and roads, for example, has been reported in mainland populations, including for *Ficus pumila* (Chen et al., 2008). Bottleneck analysis here, however, did not show any evidence for a population bottleneck on the mainland, as well as on islands excepting for BL (Table S5), indicating a relatively stable effective population size, as revealed by neutrality tests and mismatch analysis based on cpDNA. Thirdly, multiple mainland–island colonization may have occurred given that species originated on the mainland and subsequently colonized islands. This would have led to a decrease in genetic diversity with increased distance to the mainland due to the founder effect under a stepping‐stone colonization process (Yamada & Maki, 2012). However, our results did not indicate any correlation between any parameter of genetic diversity and distance to mainland. The haplotype network also did not reveal a clear divergence between mainland and islands; ancient haplotypes (H1, H3 and H7) were distributed in both regions (Figure 5) and this does not support mainland–island colonization. Fourthly, consideration of islands as refugia during the LGM (Chen et al., 2008; Fernández‐Mazuecos & Vargas, 2011; Tsumura & Ohba, 1993) can be based on relict populations frequently reported to preserve high genetic variations (García‐Verdugo et al., 2013; Hampe & Petit, 2005; Petit et al., 2003). In one example, *Cryptomeria japonica* maintained a relatively large genetic variation on Yaku Island compared with that of mainland Japan mainly because this island was refugium during the LGM and had a suitable climate (Tsumura & Ohba, 1993). In the case of the polyploid species *Prunus lusitanica*, island populations displayed more private alleles and higher genotypic diversity in old volcanic areas, suggesting the idea of oceanic islands as important refugia for biodiversity (García‐Verdugo et al., 2013). Our data also supported the hypothesis of islands as refugia in a very young, continental island system (detailed discussion seen below).

### 4.2 No effect of isolation on genetic diversity and differentiation

As proposed by the equilibrium theory of island biogeography (Losos, Ricklefs, & MacArthur, 2009; MacArthur & Wilson, 1967), larger islands can be expected to hold higher cumulative levels of diversity because of larger numbers and sizes of populations. This means that levels of genetic diversity on islands should correlate with island area (McGlaughlin et al., 2014). However, we found that island area was only a predictor for one measure of genetic diversity (*A*_R_) based on microsatellites in *M. kwangtungensis* (Figure 3), and had no significant effect on other measures either for microsatellites or cpDNA. These findings are similar to those found for two endemic island species of *Acmispon* on California Channel Islands (McGlaughlin et al., 2014), indicating that the correlation between genetic diversity and island area is not a common pattern. One possible explanation is that although a larger island is capable of sustaining more populations of a species, area should not be taken as an accurate surrogate for effective population size as this will be more significant determinant of levels of genetic diversity than number of populations (García‐Verdugo et al., 2013; McGlaughlin et al., 2014). The prediction of a positive relationship between genetic diversity and island area assumes that population densities for a species are kept the same across different islands, something that is actually unlikely to be the case. Indeed, for *M. kwangtungensis*, XX island with the smallest area was observed to have an obviously higher population density, compared to other larger islands (personal observation).

A negative relationship between distance to mainland and genetic diversity is predicted, mainly due to founder effects and limited gene flow. Although this was also seen in two previous studies on Izu Islands of Japan (Inoue & Kawahara, 1990; Yamada & Maki, 2012), we failed to establish a significant relationship in the case of *M. kwangtungensis*. The most likely explanation for this result is that island systems had different histories of formation and species colonization. In the case of the Izu Islands, these are distributed as a roughly linear chain extending from north‐to‐south near Mainland Japan and are thought to be formed southwards during the late Pleistocene through submarine volcanism (Kaneoka, Ishiki, & Zashu, 1970; Yamada & Maki, 2012), probably leading to a southward stepping stone‐like colonization from the mainland for *Weigela coraeensis* (Yamada & Maki, 2012) and *Campanula punctate* (Inoue & Kawahara, 1990). The islands studied here, in contrast, are distributed along the coast of the South China Mainland, which were connected during the LGM and were formed by rising sea levels around 7,000 years ago (Chen et al., 1993). Indeed, *M. kwangtungensis* is thought to have survived long term in these islands based on genetic and ecological niche modeling (Figure 6, see discussion below), rather than as a colonization/recolonization from a mainland source after the ice age.

Restricted land masses of islands with the geographical barrier of the sea are inclined to promote genetic differentiation among islands, and between the mainland and island populations (Frankham, 1997). Our results revealed statistically nonsignificant differences between genetic differentiations of mainland and islands at microsatellites (Figure 4, Table 3) as well as low levels of genetic differentiation between them, as indicated by the results of AMOVA for both genetic markers (Table S4). These results may be attributable to potentially high rates of gene flow (see discussion above) as well as the geographical proximity of these areas. The islands assessed here are closely distributed along the coastal line with distances to mainland ranging from 3.8 to 77.7 km and with nearest neighbors from 0.7 to 19.6 km. Thus, SM, DJ, and XX islands are within a range <6 km from the nearest peninsula (Table S1); this close proximity is likely to give rise to a high rate of gene flow, as suggested by patterns of isolation by distance (Figure 4), and the STRUCTURE analyses based on microsatellite data, where these three islands with peninsular populations (QN, YM, and DX) fell into the same cluster (Figure 2).

### 4.3 In situ glacial survival on islands

Consistent with simulated vegetation maps (Harrison et al., 2001) and sporopollen evidence (Chen et al., 1993) that a land bridge in South China provided refugia for plant species during the LGM, our findings also suggest in situ glacial survival of *M. kwangtungensis* on islands. In the first place, ENM indicates a large area with highly climatic suitability was present in regions of this land bridge during the LGM (Figure 6). Although CCSM predicted relatively limited region compared with MIROC, both verified highly suitable habitats (>0.8) on islands, implying that the current populations are partly representative of the past. Secondly, molecular evidence revealed high genetic diversity and, especially, numbers of unique haplotypes within island populations (Figure 5, Table 1). In one example, BL showed the highest level of expected heterozygosity, with a minimum sample size of eight, while XW possessed the most haplotypes and, XC and WS had the maximum haplotype diversity (Table 1). Thirdly, STRUCTURE analysis based on microsatellite data revealed two clearly genetic clusters largely separating island populations from their mainland counterparts (Figure 2), indicating that islands represent a differentiated gene pool. Based on the clusters identified by STRUCTURE, our ABC analysis indicated the highest support for the hypothesis of one refugium in islands (scenario 2) (Table 2, Figure S3), although scenario 1 cannot be completely rejected due to overlapping confidence intervals. Fourth, network of cpDNA haplotypes revealed that H7, widely distributed on islands and the mainland, were located into the interior node (Figure 5), representing the ancestral one. These patterns collectively suggest islands have acted as important refugia for *M. kwangtungensis* during the LGM and that extant island populations are the products of in situ glacial survival.

It is the case that in situ glacial refugia have been frequently reported for temperate (Parisod & Besnard, 2007; Zeng et al., 2015) and evergreen broad‐leaved forests (Chen et al., 2020; Gong et al., 2016; Wang et al., 2015), and mountainous regions have usually been regarded as critical glacial refugia because their high topographical heterogeneity offers scope for shifts in altitudinal range in response to climate changes (Shi et al., 2014). Islands, due to their stable climate and environmental heterogeneities, also have the potentials for in situ glacial survival of species, as evidenced by accumulating studies (García‐Verdugo et al., 2013, 2019; Hutsemékers et al., 2011). In South China, land bridge, connecting between continental islands and mainland during glacial periods, may provide suitable habitats with a minor shift of temperature and humidity (Chen et al., 2012; Weigelt et al., 2013), and have supported effective population sizes for in situ persistence, as indicated in Hainan Island (Chang et al., 2012) and Taiwan Island (Cheng, Hwang, & Lin, 2005).

### 4.4 Demographic history

Typical responses of plants to climate changes include geographical migration, resulting in the alteration of distribution range (Etterson & Shaw, 2001). The current distribution of *M. kwangtungensis* is mainly restricted within a narrow belt in southern Guangdong Province. Based on ENM reconstruction, the possible occurrence of populations during the LGM mainly covered a greater area extending current distribution to southern land bridges (Figure 6), indicating much more suitable habitats for survival. Thereby, the growth of population size is considered to be expected. Our genetic evidence supported this idea: significantly negative *D* and *F*s, unimodality of mismatch distribution (Table 5), and a slightly ascending curve for BSP (Figure S4) for all populations and island cluster were evident for population expansion (10,700–32,200 years BP), while inland populations kept a relatively stable population size. It is plausible that when climate cooling, land bridge connected islands to mainland, facilitating island populations expanding in space and population size, which may appear to be an unusual scenario, different from the general pattern of demographic contraction in glacial periods, but population growth in response to glaciation‐induced increase in lowland area has recently been suggested for wide variety of species (Chen et al., 2012; Nakamura et al., 2014).

ABC analysis indicated the highest support for the hypothesis that there was one refugium in islands and inland populations were derived from island at around 3,930 years ago, when considering a generation time of 10 years for *M. kwangtungensis* (scenario 2) (Table 2, Figure S3). This scenario should be treated with caution given the overlapping confidence intervals with scenario 1 (one refugia in inland and island derived from inland). Since hypothesis of more than two refugia was rejected (Table 2, Fig. S3), one continuous refugia, where inland and island situated, may be an acceptable hypothesis, as predicted by both CCSM and MIROC during LGM (Figure 6). Both scenario 1 and scenario 2 supported a divergence time of ca. 3,000–4,000 years ago, compatible with the time of island formation, implying that vicariance sundered to the continuous distribution range of ancestral lineages (Nakamura et al., 2010).

## 5 CONCLUSIONS

The molecular data presented here for *M. kwangtungensis*, an endemic species to South China with a narrow distribution, revealed a similar level of genetic diversity in islands compared with the mainland, contradictory with the general expectation that insular populations often harbor lower genetic variation due to founder effects and geographical isolation (Frankham, 1997). The genetic diversity pattern of this species may be explained by close proximity and a potentially high rate of gene flow among populations as well as by historical processes. It is possible that current islands acted as refugia during the LGM and that extant island populations experienced in situ glacial survival, confirmed by differentiated lineages and high levels of polymorphism, together with the strong support of a suitable climate. Our results highlight a key role of in situ glacial survival in maintaining high levels of genetic diversity in continental islands across southern subtropical China. This result means that these islands provide a model system for future studies to assess dispersal, divergence, and the ecological adaptation of insular species in a young and adjacent mainland–island system.

## CONFLICT OF INTEREST

None declared.

## AUTHOR CONTRIBUTION

**Miao‐Miao Shi:** Conceptualization (lead); Data curation (lead); Formal analysis (lead); Funding acquisition (equal); Methodology (lead); Software (equal); Writing‐original draft (lead); Writing‐review & editing (equal). **Yuyuan Wang:** Formal analysis (equal); Investigation (equal). **Tingting Duan:** Methodology (equal); Software (equal). **Xin Qian:** Formal analysis (equal); Visualization (equal). **Tong Zeng:** Methodology (equal); Software (equal); Visualization (equal). **Dian‐Xiang Zhang:** Conceptualization (equal); Funding acquisition (equal); Supervision (equal); Writing‐review & editing (equal).

## Supporting information

Appendix S1

Appendix S2

## Data Availability

Haplotype sequences were deposited in GenBank under the accession numbers MT349425–MT349442, and MT361334–MT361343. An aligned sequence matrix and microsatellite data can be found at Dryad Digital Repository: https://doi.org/10.5061/dryad.r4xgxd294.
