# Supplementary material for: In situ glacial survival maintains high genetic diversity of Mussaenda kwangtungensis on continental islands in subtropical China
Source: Ecol Evol. 2020 Sep 17;10(20):11304–21. doi: 10.1002/ece3.6768 (PMC7593160; doi:10.1002/ece3.6768)
Supplement: Supplementary file 1 — Appendix S1 [file ECE3-10-11304-s001.docx]

**SUPPORTING INFORMATION – Appendix S1 for:**

*In situ* glacial survival maintains high genetic diversity of *Mussaenda kwangtungensis* on continental islands in subtropical China

Miaomiao Shi, Yuyuan Wang, Tingting Duan, Xin Qian, Tong Zeng, Dianxiang Zhang

**S1.1** **Laboratory protocols for microsatellites and cpDNA sequencing.**

**Microsatellites**: Multiplex polymerase chain reactions (PCR) were performed in a 10 *μ*L mixture containing 5 *μ*L Master Mix (Tiangen, Guangzhou, China), 0.2 *μ*M of each primer pair, 3.6 *μ*L deionized water, and 30–50 ng of genomic DNA. CT99 (Label: TAMRA), CT142 (ROX), CT48 (FAM) and CAA92 (HEX) were in the first PCR amplification. CT59 (ROX), CT135 (HEX) and AC30 (FAM) were in the second PCR amplification. CT17 (ROX), CT113 (FAM) and CT12 (HEX) were in the third PCR amplification. The last single PCR amplification was performed for CT60 (HEX). Thermocycler protocol were initial denaturing at 94℃ for 4 min, then 35 cycles at 94℃ for 30 s, 30 s at annealing temperature (Duan & Zhang, 2014), and 30 s at 72℃, with a final extension of 8 min at 72℃.

**cpDNA**: The PCR was performed in a 25 *μ*L mixture containing 12.5 *μ*L Master Mix (Tiangen, Guangzhou, China), 0.3 μM of each primer pair, 10.5 μL deionized water, and 30–50 ng of genomic DNA. A touchdown PCR programme was used with initial denaturation for 4 min at 94℃, 94℃ for 30 s, 60℃ for 30 s and 72℃ for 30 s, followed 7 cycles at decreasing annealing temperatures in decrements of 1℃ per cycle, then followed by 94℃ for 30 s, 30 s at 53℃, 30 s at 72℃, ended with an extra extension of 8 min at 72℃.

Table S1 Population locations and characteristics of islands in this study.

| Site | code | Location | Area (km^2^) | DTM (km) | DTI  (km) | Altitude  (m) |
| --- | --- | --- | --- | --- | --- | --- |
| Naobeishan | NB | 22.114 N, 113.531 E |  |  |  |  |
| Gudoushan | GD | 22.196 N, 112.947 E |  |  |  |  |
| Zhuguangshan | ZG | 22.209 N, 113.478 E |  |  |  |  |
| Jiangjunshan | JJ | 22.246 N, 113.578 E |  |  |  |  |
| Banzhangshan | BZ | 22.248 N, 113.550 E |  |  |  |  |
| Shijingshan | SJ | 22.260 N, 113.582 E |  |  |  |  |
| Zhuhaimishui | ZM | 22.353 N, 113.568 E |  |  |  |  |
| Xiangtoushan | XT | 23.279 N, 114.426 E |  |  |  |  |
| Qiniangshan | QN | 22.495 N, 114.584 E |  |  |  |  |
| Yangmeikeng | YM | 22.547 N, 114.576 E |  |  |  |  |
| Daxingshan | DX | 22.557 N, 114.895 E |  |  |  |  |
| Xiachuandao | XC | 21.656 N, 112.606 E | 98.69 | 6.6 | 4.3 | 542 |
| Shangchuandao | SC | 21.701 N, 112.772 E | 157.00 | 11.8 | 4.3 | 450 |
| Wanshandao | WS | 21.934 N, 113.727 E | 8.18 | 21.7 | 0.7 | 432.5 |
| Xiaowanshandao | XW | 21.951 N, 113.693 E | 5.71 | 18.7 | 0.7 | 250.7 |
| Bailidao | BL | 21.986 N, 113.759 E | 8.03 | 21.4 | 2.4 | 299.3 |
| Dong’aodao | DA | 22.017 N, 113.706 E | 4.65 | 15.8 | 3.0 | 169 |
| Dangandao | DG | 22.053 N, 114.301 E | 13.49 | 77.7 | 19.2 | 322 |
| Wailingtingdao | WL | 22.103 N, 114.033 E | 4.31 | 48.4 | 19.2 | 311.8 |
| Guishandao | GS | 22.133 N, 113.822 E | 3.60 | 26.9 | 14.6 | 233.5 |
| Sanmendao | SM | 22.463 N, 114.629 E | 4.79 | 3.8 | 10.7 | 298 |
| Xiaoxingshandao | XX | 22.514 N, 114.841 E | 1.27 | 4.6 | 19.6 | 118 |
| Dajiadao | DJ | 22.578 N, 114.646 E | 1.80 | 5.7 | 10.7 | 111.6 |

DTM, the nearest distance to mainland; DTI, distance to the nearest islands where *Mussaenda kwangtungensis* distributes.

**Table S2** Occurrence records of *Mussaenda kwangtungensis* used for modelling species distribution in the past.

| Code | Site | Latitude (N) | Longitude  (E) | Voucher collector/no. |
| --- | --- | --- | --- | --- |
| 1 | Nankunshan, Guangdong Province | 23.641 | 113.885 | DTT747 |
| 2 | Shangchuandao, Guangdong Province | 21.701 | 112.772 | ZLX11742 |
| 3 | Xiachuandao, Guangdong Province | 21.656 | 112.606 | ZLX11567 |
| 4 | Xiaoxingshandao, Guangdong Province | 22.514 | 114.841 | WYY10 |
| 5 | Bailidao, Guangdong Province | 21.986 | 113.759 | HXX16695 |
| 6 | Wanshandao, Guangdong Province | 21.934 | 113.727 | HXX16586 |
| 7 | Xiaowanshandao, Guangdong Province | 21.951 | 113.693 | HXX16792 |
| 8 | Dajiadao, Guangdong Province | 22.578 | 114.646 | WYY25 |
| 9 | Dong’aodao, Guangdong Province | 22.017 | 113.706 | HXX16527 |
| 10 | Sanmendao, Guangdong Province | 22.463 | 114.629 | WYY28 |
| 11 | Dangandaotou, Guangdong Province | 22.053 | 114.301 | WYY32 |
| 12 | Dangandaowei, Guangdong Province | 22.033 | 114.244 | WYY31 |
| 13 | Wailingdingdao, Guangdong Province | 22.103 | 114.033 | WYY30 |
| 14 | Guishandao, Guangdong Province | 22.133 | 113.822 | HXX16906 |
| 15 | Xiangtoushan, Guangdong Province | 23.279 | 114.426 | WYY4 |
| 16 | Banzhangshan, Guangdong Province | 22.248 | 113.550 | WYY7 |
| 17 | Mishui, Guangdong Province | 22.353 | 113.568 | WYY22 |
| 18 | Naobeishan, Guangdong Province | 22.114 | 113.531 | WYY20 |
| 19 | Shijingshan, Guangdong Province | 22.246 | 113.578 | WYY19 |
| 20 | Jiangjunshan, Guangdong Province | 22.246 | 113.578 | WYY18 |
| 21 | Zhuguangshan, Guangdong Province | 22.209 | 113.478 | WYY16 |
| 22 | Gudoushan, Guangdong Province | 22.196 | 112.947 | DTT665 |
| 23 | Qiniangshan, Guangdong Province | 22.495 | 114.584 | DTT817 |
| 24 | Daxingshan, Guangdong Province | 22.557 | 114.895 | WYY13 |
| 25 | Yangmeikeng, Guangdong Province | 22.547 | 114.576 | WYY15 |
| 26 | Liuxihe Nature Reserve, Guangdong Province | 23.745 | 113.784 | DXF722524 |
| 27 | Qimuzhang Nature Reserve, Guangdong Province | 23.799 | 115.446 | LXG265913 |
| 28 | Hongkong | 22.409 | 114.138 | CHY8597 |

All voucher specimen were deposited in South China Botanical Garden, Chinese Academy of Sciences.
