# Supplementary material for: In situ glacial survival maintains high genetic diversity of Mussaenda kwangtungensis on continental islands in subtropical China
Source: Ecol Evol. 2020 Sep 17;10(20):11304–21. doi: 10.1002/ece3.6768 (PMC7593160; doi:10.1002/ece3.6768)

**SUPPORTING INFORMATION – Appendix S2 for:**

*In situ* glacial survival maintains high genetic diversity of *Mussaenda kwangtungensis* on continental islands in subtropical China

Miaomiao Shi, Yuyuan Wang, Tingting Duan, Xin Qian, Tong Zeng, Dianxiang Zhang

Table S3 Prior distributions of the parameters used in DIYABC.

| Parameter | Minimum | Maximum |
| --- | --- | --- |
| Effective population size |  |  |
| N1 | 10 | 10000 |
| N2 | 10 | 10000 |
| N3 | 10 | 10000 |
| Na | 10 | 15000 |
| Time scale in generations |  |  |
| t1 | 10 | 10000 |
| t2 | 50 | 10000 |
| t3 | 10 | 10000 |
| Admixture rate |  |  |
| ra | 0.001 | 0.999 |
| Mutation model |  |  |
| Mean mutation rate | 1.00E-04 | 1.00E-03 |
| Individual locus mutation rate | 1.00E-05 | 1.00E-02 |
| Mean coefficient P | 1.00E-01 | 3.00E-01 |
| Individual locus coefficient P | 1.00E-02 | 9.00E-01 |
| Mean SNI rate | 1.00E-08 | 1.00E-05 |
| Individual locus SNI rate | 1.00E-09 | 1.00E-04 |

Table S4 Analysis of molecular variance (AMOVA) based on microsatellites and cpDNA data.

| Source of variation | d.f. | Sum of squares | Variance components | Percentage of variation (%) |
| --- | --- | --- | --- | --- |
| Microsatellites |  |  |  |  |
| Among groups | 1 | 45.670 | 0.038 | 0.90 |
| Among populations within groups | 21 | 443.160 | 0.322 | 7.58 |
| Within populations | 1211 | 4714.224 | 3.893 | 91.52 |
| Total | 1233 | 5203.053 | 4.253 |  |
| cpDNA |  |  |  |  |
| Among groups | 1 | 3.321 | 0.004 | 0.34 |
| Among population within groups | 21 | 59.794 | 0.442 | 39.86 |
| Within populations | 92 | 60.955 | 0.663 | 59.81 |
| Total | 114 | 124.070 | 1.108 |  |

Table S5 Bottleneck tests for each population of *Mussaenda kwangtungensis*.

| Population | TPM | SMM | Mode shift |
| --- | --- | --- | --- |
| mainland |  |  |  |
| NB | 0.989 | 0.999 | L-shaped |
| GD | 0.840 | 0.992 | L-shaped |
| ZG | 0.992 | 0.999 | L-shaped |
| JJ | 0.416 | 0.840 | L-shaped |
| BZ | 0.382 | 0.989 | L-shaped |
| SJ | 0.382 | 0.966 | L-shaped |
| ZM | 0.995 | 1.000 | L-shaped |
| XT | 0.949 | 0.999 | L-shaped |
| QN | 0.959 | 0.999 | L-shaped |
| YM | 0.966 | 0.999 | L-shaped |
| DX | 0.997 | 1.000 | L-shaped |
| island |  |  |  |
| XC | 0.861 | 0.989 | L-shaped |
| SC | 0.650 | 0.995 | L-shaped |
| WS | 0.681 | 0.992 | L-shaped |
| XW | 0.938 | 0.998 | L-shaped |
| BL | 0.183 | 0.350 | Shifted |
| DA | 0.840 | 0.994 | L-shaped |
| DG | 0.995 | 0.999 | L-shaped |
| WL | 0.966 | 1.000 | L-shaped |
| GS | 0.711 | 0.992 | L-shaped |
| SM | 0.740 | 0.999 | L-shaped |
| XX | 0.926 | 0.995 | L-shaped |
| DJ | 0.938 | 0.997 | L-shaped |

TPM and SMM, probability of one tail for heterozygosity excess under two-phase model (TPM) and step-mutation model (SMM).

Table S6 Chloroplast DNA sequence variation in three fragments identifying 21 haplotypes (H1-H21).

|  | psbE-petL | | | | | | | | | | | | rpl32-trnL | | | | | | | | ndhf-rpl32 | | | | | | | |
| --- | --- | --- | --- | --- | --- | --- | --- | --- | --- | --- | --- | --- | --- | --- | --- | --- | --- | --- | --- | --- | --- | --- | --- | --- | --- | --- | --- | --- |
|  | 2  0  7 | 3  3  7 | 4  0  3 | 4  5  7 | 6  7  5 | 7  4  8 | 8  2  3 | 8  5  1 | 8  5  8 | 9  5  9 | 1  1  3  4 | 1  1  7  6 | 2  1  2 | 2  2  9 | 3  5  6 | 4  7  5 | 5  8  6 | 6  6  3 | 6  8  0 | 8  6  0 | 9  9 | 8  0  0 | 8  1  8 | 8  3  3 | 8  3  8 | 8  4  2 | 8  4  6 | 1  0  3  1 |
| H1 | A | A | C | A | C | G | T | # | C | C | T | G | G | C | A | T | A | T | C | A | T | A | C | C | A | G | $ | A |
| H2 | A | A | C | A | C | G | T | # | A | C | T | T | G | C | A | T | A | T | C | A | T | A | C | C | A | G | $ | A |
| H3 | A | A | C | A | C | G | T | # | C | C | T | T | G | A | A | T | A | T | C | A | T | A | C | C | A | G | $ | A |
| H4 | A | A | C | A | C | G | T | # | C | C | T | T | G | A | A | T | G | T | C | A | T | A | C | C | A | G | $ | A |
| H5 | A | A | C | A | C | G | T | # | C | C | T | T | G | A | A | T | G | T | C | A | T | A | C | A | A | G | $ | A |
| H6 | G | A | C | A | C | G | T | # | C | C | T | G | G | C | A | T | A | T | C | A | T | A | C | C | A | G | $ | A |
| H7 | A | A | C | A | C | G | T | # | C | C | T | T | G | C | A | T | A | T | C | A | T | A | C | C | A | G | $ | A |
| H8 | A | A | C | A | C | G | T | # | C | C | T | T | G | A | A | T | A | T | C | A | T | A | C | C | T | G | $ | A |
| H9 | A | A | C | A | C | G | T | # | C | C | T | G | G | C | A | T | A | T | C | A | T | A | C | C | A | T | $ | A |
| H10 | A | A | C | A | C | G | T | # | C | C | T | G | G | C | A | T | A | C | C | A | T | A | C | C | A | G | $ | A |
| H11 | A | A | C | G | A | G | T | — | C | C | T | T | T | A | A | T | A | T | C | G | T | A | T | C | A | G | $ | A |
| H12 | A | A | C | A | C | G | T | — | C | C | T | G | G | C | A | T | A | T | C | A | T | A | C | C | A | G | $ | A |
| H13 | A | C | A | A | C | T | T | # | C | C | T | T | G | C | A | T | A | T | C | A | T | A | C | C | A | G | $ | A |
| H14 | A | A | C | A | C | G | T | # | C | C | T | T | G | C | A | T | A | T | A | A | T | A | C | C | A | G | $ | A |
| H15 | A | A | C | A | C | G | T | # | C | C | T | T | G | C | T | T | A | T | C | A | T | A | C | C | A | G | $ | A |
| H16 | A | A | C | A | C | G | T | # | C | C | C | G | G | C | A | T | A | T | C | A | C | G | C | C | A | G | $ | A |
| H17 | A | A | C | A | C | G | T | # | C | A | T | T | G | A | A | T | A | T | C | A | T | A | C | C | T | G | $ | A |
| H18 | A | A | C | A | C | G | T | # | C | A | T | T | G | A | A | T | A | T | C | A | T | A | C | C | A | G | $ | A |
| H19 | A | A | C | A | C | G | G | # | C | C | T | T | G | A | A | G | A | T | C | A | T | A | C | C | A | G | $ | A |
| H20 | A | A | C | A | C | G | G | # | C | C | T | T | G | A | A | G | A | T | C | A | T | A | C | C | A | G | $ | C |
| H21 | A | A | C | A | C | G | T | # | C | C | T | T | G | A | T | T | A | T | C | A | T | A | C | C | A | G | — | A |

#CAAAATT; $CTTATCT

Fig. S1 The seven scenarios tested in DIYABC. In these scenarios, t# represents the time-scale in terms of the number of generations, N# represents the effective populations size of each corresponding gene pool, and ra represents admixture rate.


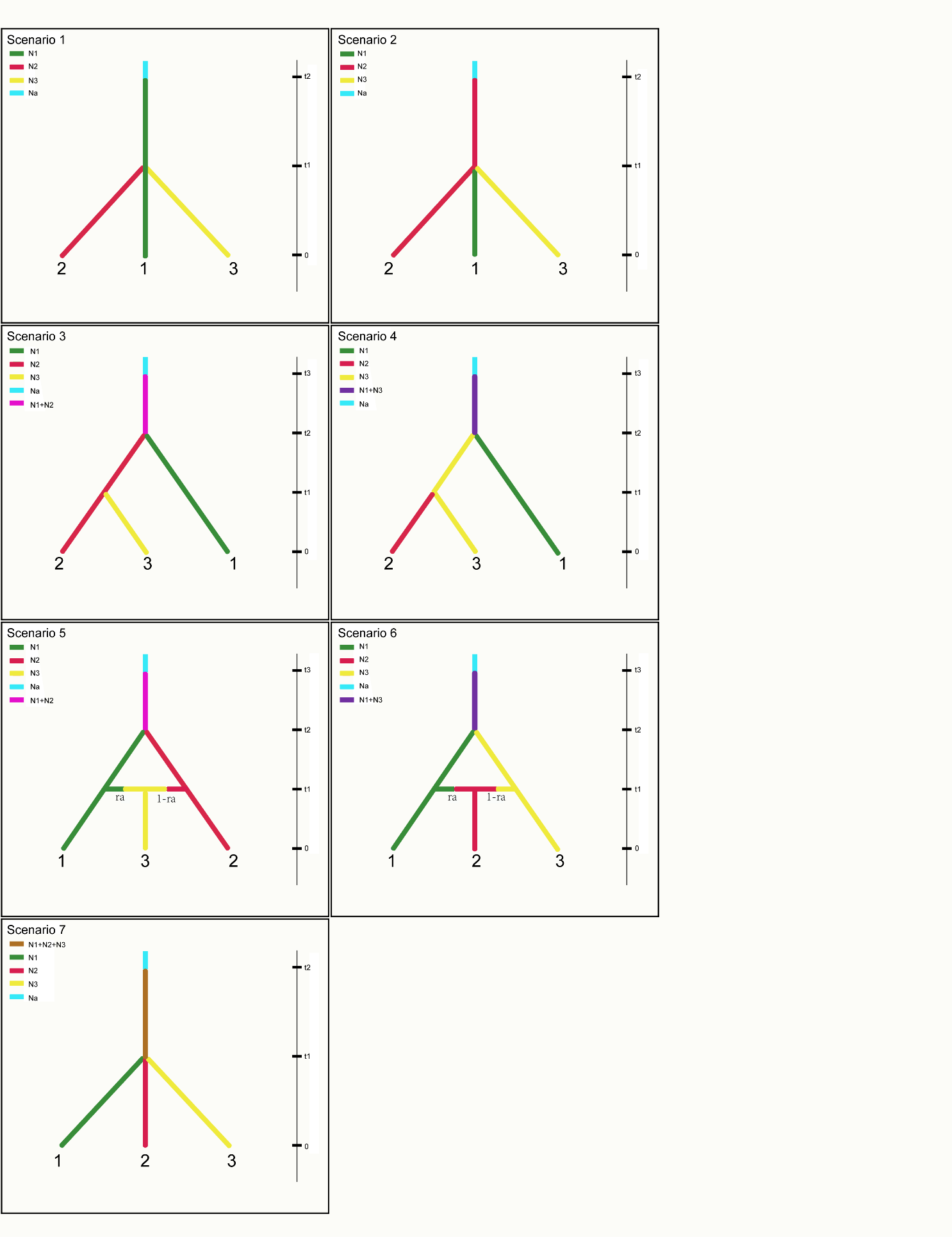


Fig. S2 Inference of K, the most probable number of clusters, using STRUCTURE software. (A) Log-likelihood value of data, Ln P(*K*), as a function of *K* for ten replicates; (B) Dleta *K* as a function of *K* calculated over ten replicates.


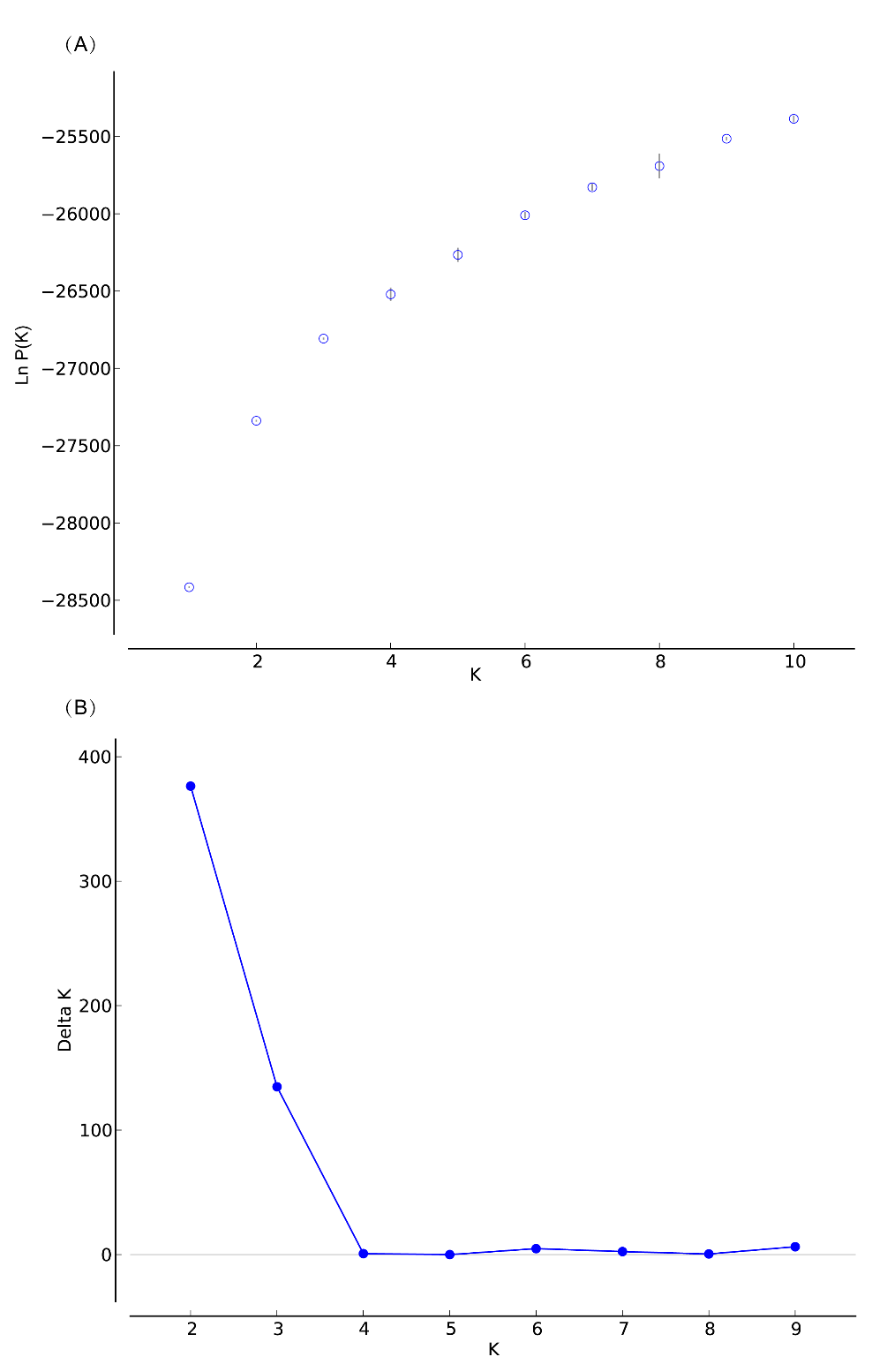


Fig. S3 Logistic regression of the posterior probabilities associated to each scenario of divergence of *Mussaenda kwangtungensis* populations, as computed in DIYABC.


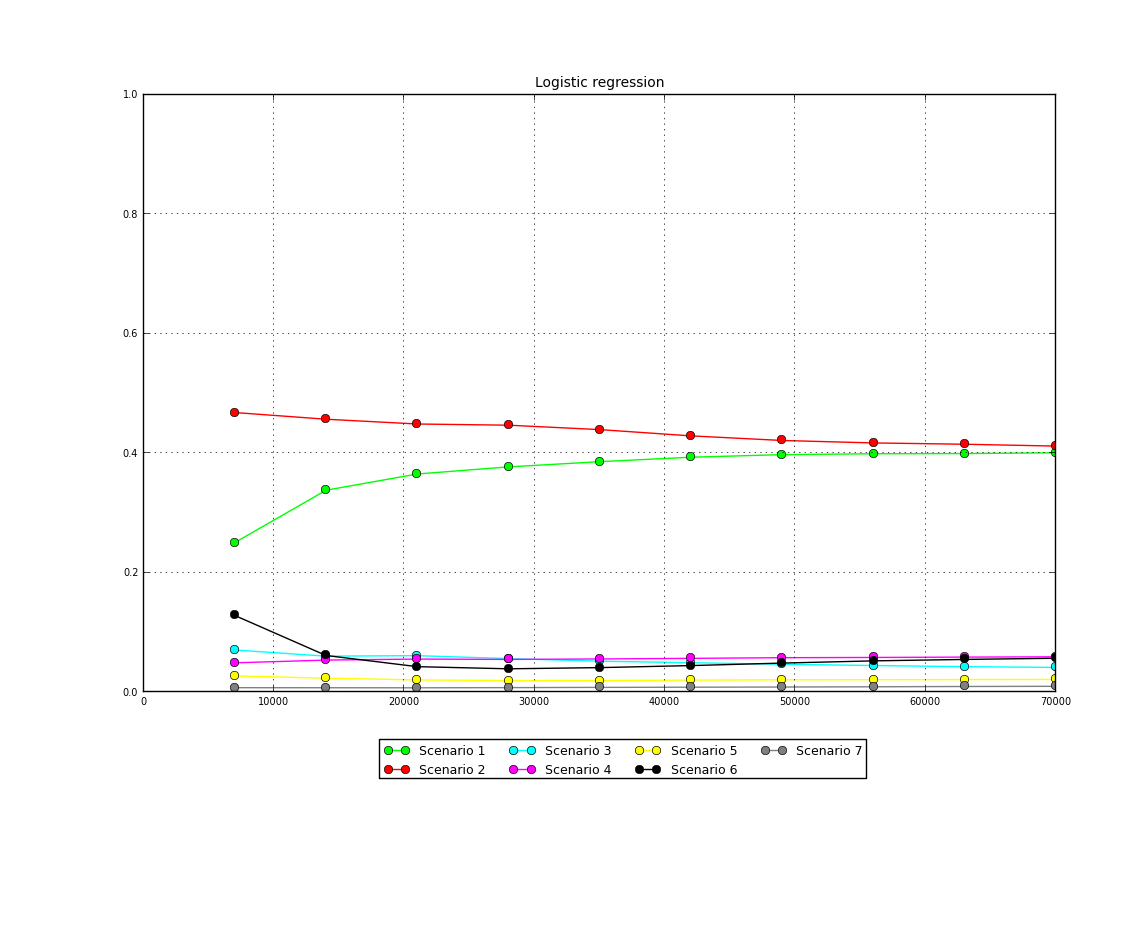


Fig. S4 Bayesian skyline plots for (A) all populations, (B) island cluster and (C) inland cluster, showing effective population size as a function of time (years B). The upper and lower limits of light blue trend represent the 95% confidence intervals.


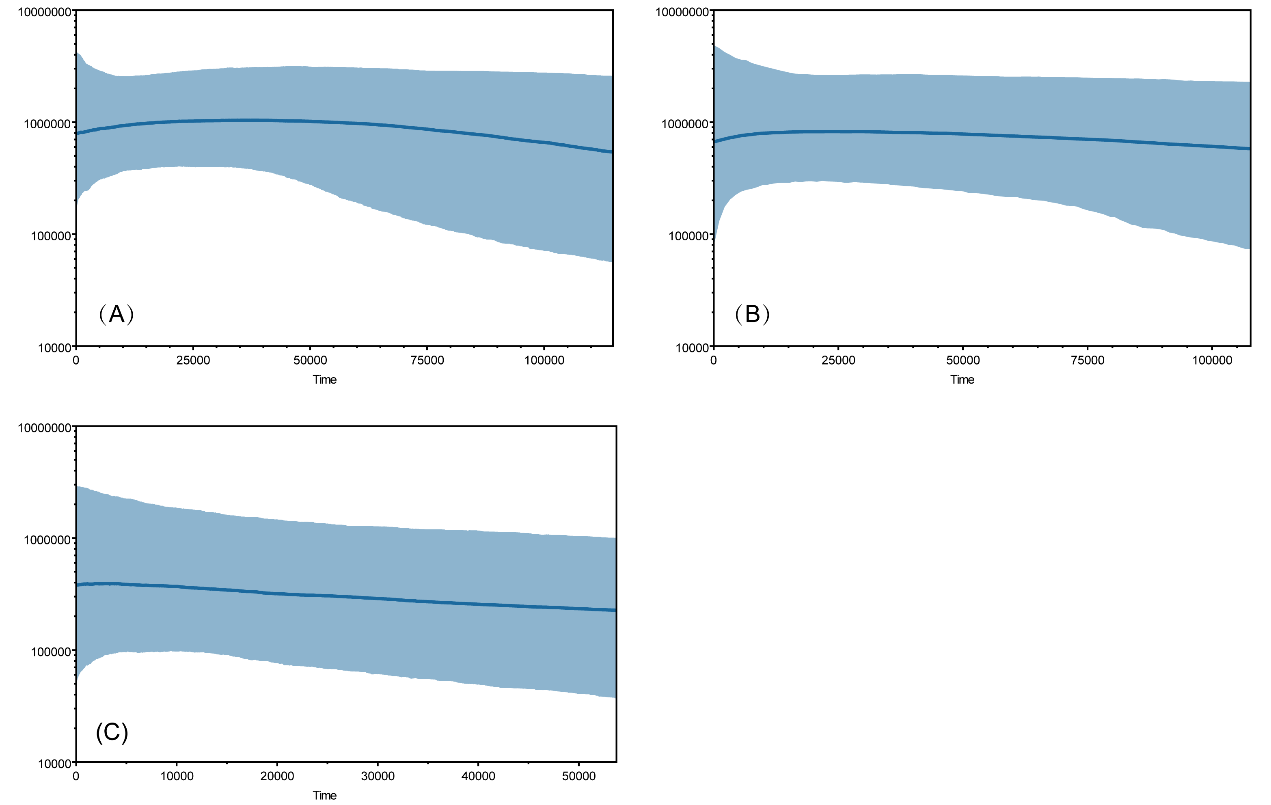

Supplement: Supplementary file 2 — Appendix S2 [file ECE3-10-11304-s002.docx]
